# Generation of universal and hypoimmunogenic human pluripotent stem cells

**DOI:** 10.1111/cpr.12946

**Published:** 2020-11-11

**Authors:** Qingsong Ye, Tzu‐Cheng Sung, Jen‐Ming Yang, Qing‐Dong Ling, Yan He, Akon Higuchi

**Affiliations:** ^1^ School and Hospital of Stomatology Wenzhou Medical University Wenzhou China; ^2^ Center of Regenerative Medicine Renmin Hospital of Wuhan University Wuhan China; ^3^ Skeletal Biology Research Center Department of Oral Maxillofacial Surgery Massachusetts General Hospital & Harvard School of Dental Medicine Boston MA USA; ^4^ School of Ophthalmology and Optometry Eye Hospital Wenzhou Medical University Wenzhou China; ^5^ Department of Chemical and Materials Engineering National Central University Taoyuan Taiwan; ^6^ Department of Chemical and Materials Engineering Chang Gung University Taoyuan Taiwan; ^7^ Cathay Medical Research Institute, Cathay General Hospital Taipei Taiwan; ^8^ Wenzhou Institute University of Chinese Academy of Science Wenzhou China; ^9^ Department of Chemical Engineering and R&D Center for Membrane Technology Chung Yuan Christian University Taoyuan Taiwan; ^10^ Center for Emergent Matter Science, Riken Saitama Japan

**Keywords:** regenerative medicine, stem cells

## Abstract

There is a need to store very large numbers of conventional human pluripotent stem cell (hPSC) lines for their off‐the‐shelf usage in stem cell therapy. Therefore, it is valuable to generate “universal” or “hypoimmunogenic” hPSCs with gene‐editing technology by knocking out or in immune‐related genes. A few universal or hypoimmunogenic hPSC lines should be enough to store for their off‐the‐shelf usage. Here, we overview and discuss how to prepare universal or hypoimmunogenic hPSCs and their disadvantages. β2‐Microglobulin‐knockout hPSCs did not harbour human leukocyte antigen (HLA)‐expressing class I cells but rather activated natural killer (NK) cells. To avoid NK cell and macrophage activities, homozygous hPSCs expressing a single allele of an HLA class I molecule, such as HLA‐C, were developed. Major HLA class I molecules were knocked out, and PD‐L1, HLA‐G and CD47 were knocked in hPSCs using CRISPR/Cas9 gene editing. These cells escaped activation of not only T cells but also NK cells and macrophages, generating universal hPSCs.

## Graphical abstract

To avoid natural killer (NK) cell and macrophage activities, homozygous human pluripotent stem cells (hPSCs) expressing a single allele of an HLA class Ia molecule, such as HLA‐C, were developed (left picture). Major HLA class I molecules were knocked out and PD‐L1, HLA‐G and CD47 were knocked in hPSCs using CRISPR/Cas9 gene editing (right picture). These cells escaped activation of not only T cells but also NK cells and macrophages, generating universal hPSCs.

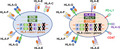

## 1 INTRODUCTION

**Figure 1:**
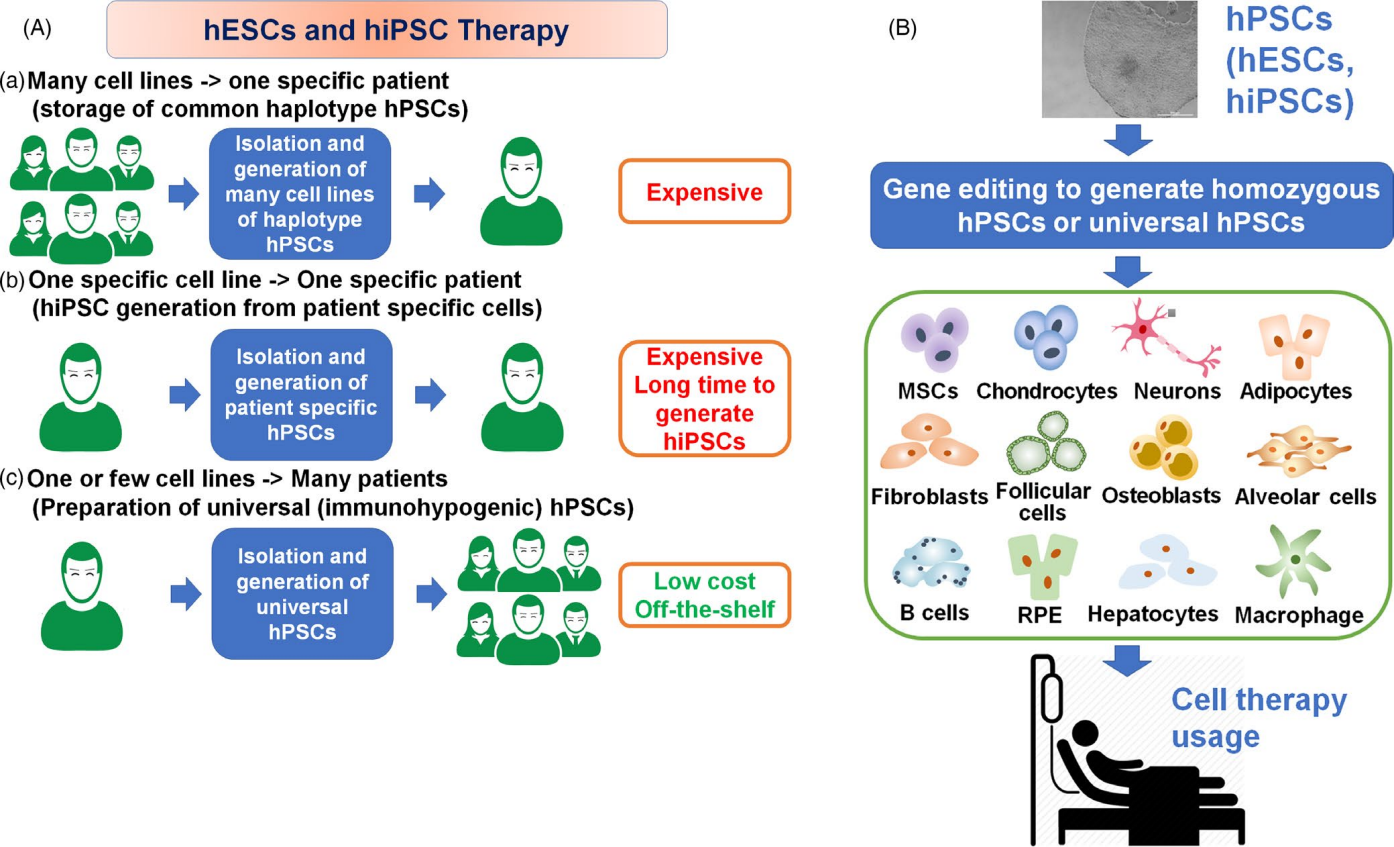
Human ESC and hiPSC therapy. A, (a) A single cell line matched to a specific patient's HLA type is selected among many hESC and hiPSC lines, which will be utilized for cell therapy in a specific patient. (b) Patient cells are collected and reprogrammed into patient‐specific hiPSCs, which will be utilized for cell therapy in a specific patient. (c) A few lines of hypoimmunogenic or universal hESCs and hiPSCs are stored, which will be utilized for cell therapy in any patient. B, Overview of stem cell therapy using universal hESCs and hiPSCs. Wild‐type hESCs and hiPSCs are prepared to be hypoimmunogenic or universal hESCs and hiPSCs by knocking out certain HLA genes and/or knocking in immune‐related genes. Then, universal hESCs and hiPSCs are differentiated into specific cell types and subsequently used for cell therapy to treat patients

Millions of humans suffer from the loss and injury of organs and tissues each year from diseases, accidents and birth defects. Stem cells are considered a valuable source for regenerative medicine through cell therapy.^1^, ^2^, ^3^, ^4^, ^5^ Human pluripotent stem cells (hPSCs), such as human induced pluripotent stem cells (hiPSCs) and human embryonic stem cells (hESCs), are able to differentiate into any type of cell in the human body.^6^, ^7^, ^8^, ^9^, ^10^ Subsequently, the differentiated cells from hPSCs can be utilized as the cell source for patient therapies (Figure 1). However, there are immunological barriers to the clinical usage of hPSCs that restrict the transplantation of allogeneic cells. The immunological barriers originate from the matching of human leukocyte antigens (HLAs) on the cell surface between patients and allogeneic transplanted cells. The HLA genes are encoded on chromosome 6 as the major histocompatibility complex (MHC) and harbour foreign peptides and self‐peptides to T cells. These polymorphic loci are categorized as the major class I genes (class Ia; HLA‐A, ‐B and ‐C), which are expressed on typical nucleated cells, and the major class II genes (HLA‐DP, ‐DQ and ‐DR), which are expressed in T cells, B cells and antigen‐presenting cells (macrophages and dendritic cells). Furthermore, class II expression can be induced in other cell types upon stimulation by interferons.^11^ Each polymorphic HLA gene harbours very large numbers of multiple alleles (Table 1),^12^ and therefore, it is extremely difficult to find a cell, organ or tissue donor who matches a specific pair of HLA alleles. Typically, cells, tissues and organs with mismatched HLA haplotypes are transplanted into patients treated with immunosuppressive therapy. However, prolonged treatment with immunosuppressive medicine, which is required to prevent the rejection of mismatched cells and grafts, often leads to dangerous side effects.

**Table 1:** Variation of HLA class I and class II alles

Gene	No. of Alles
HLA class I (major)	
HLA‐A	5266
HLA‐B	6537
HLA‐C	5140
HLA class I (minor)	
HLA‐E	43
HLA‐F	44
HLA‐G	69
HLA class II	
HLA‐DR	3168
HLA‐DQ	1901
HLA‐DP	1581
HLA‐DM	20
HLA‐DO	25

The disadvantage of hPSC therapies is that many types of hPSCs with specific HLA class I and class II molecules need to be stocked for their banking for hPSC therapies (Figure 1A(a)). Taylor and colleagues suggested that the development of approximately 150 hESC lines with different HLA types would be necessary to provide sufficient HLA types to match hESC derivatives for most patients in the UK.^13^ Nakajima and colleagues suggested that 170 hESC lines with different HLA types would support HLA matching for 80% of patients in Japan (but not 100% of patients).^14^ However, more than 150 000 donors must be screened to generate 140 HLA‐A, ‐B and ‐DR homozygous hPSC lines.^11^ The extremely high cost needed to store large numbers of hPSC lines, even 150‐170 cell lines, for their transplantation will lead to a deficit in the national insurance budget if hPSC therapies are approved for usage by national insurance in every country.

Recently, hPSCs, which have approximately the same characteristics as hESCs, were generated from adult somatic cells through the “forced” expression of (a) certain pluripotent genes,^15^ such as *Sox2*, *Oct3/4*, *klf‐4* and *l‐myc* or *c‐myc*, (b) proteins (protein‐induced PSCs)^16^, ^17^ or (c) miRNAs,^16^, ^18^ which can be prepared from patient cells.^16^ These cells were named hiPSCs. However, one of the difficulties associated with the application of hiPSCs for patient‐specific therapy is that much time is required to generate mature and clinical‐grade hiPSCs because they require an evaluation to prove that there is no contamination with viruses or other pathogens and no genetic abnormality, contributing to the high cost associated with hiPSC therapies and the time‐consuming and laborious procedures (Figure 1A(b)). However, patient‐specific hiPSCs have no immunogenicity‐related problems after the transplantation of differentiated cells from these hiPSCs because hiPSCs have identical HLA haplotypes.

## 2 PREPARATION METHODS OF UNIVERSAL (HYPOIMMUNOGENIC) HPSCS

It is necessary to develop hPSCs that do not express HLA class I and class II molecules (universal hPSCs) even after differentiation into specific cell lineages. These universal or hypoimmunogenic hPSCs can be used for clinical therapy in patients with different types of HLA class I and class II molecules (Figure 1A(c) and B). There are several strategies that can be used to reduce the number of banked hPSC lines: (a) generation of universal hPSCs by knocking out β2‐microglobulin (B2M), (b) generation of HLA‐homozygous hPSCs by gene editing and (c) generation of universal or hypoimmunogenic hPSC by knocking in specific genes. We will discuss these methods, and we will focus on the advantages and disadvantages of these methods in preparing universal and hypoimmunogenic hPSCs. Table 2 summarizes some studies that have generated universal and hypoimmunogenic hPSCs.^5^, ^35^

**Table 2:** Preparation methods to make universal and hypoimmunogenic hiPSCs

Cell	Knocking out B2M gene	Knocking out genes except B2M	Knocking in genes	Cell culture biomaterials	Differentiation lineages	Ref.
Knocking out B2M gene						
hESCs (H1, H7, BG01, BG02, BG03)	B2M knocking out	—	—	Not described	EB	[21] (2013)
hiPSCs	B2M knocking out using TALEN technology	—	—	Matrigel, Col IV	Platelets, megakaryocytes	[22] (2014)
hESCs (RUES2)	B2M knocking out using shRNA	—	—	Vitronectin	Cardiomyocytes, hepatic lineages	[23] (2015)
hESCs (H9.2)	B2M knocking out	—	—	MEF, Matrigel	Lung alveolar epithelial type II cells	[19] (2015)
hiPSCs	B2M knocking out using shRNA	—	—	LN‐521	Platelets, Megakaryocytes	[24] (2016)
hESCs (Elf‐1, H1, H9)	B2M knocking out	—	Knocking in HLA‐E	Matrigel, gelatin	RPE, EB, CD45^+^ hematopoietic cells	[27] (2017)
HiPSCs	B2M knocking out	Knocking out CIITA	—	Matrigel	Cardiomyocytes	[26] (2018)
hiPSCs	B2M knocking out	—	—	MEF	HSCs, platelets, megakaryocytes	[25] (2019)
hiPSCs	B2M knocking out	Knocking out CIITA	Knocking in CD47	Matrigel	Endothelial cells, cardiomyocytes	[5] (2019)
hiPSCs (strategy 2)	B2M knocking out	—	Knocking in HLA‐C, HLA‐E, HLA‐G & HLA‐F)	iMatrix‐511	CD43^+^ blood cells, cardiomyocytes	[11] (2019)
hESCs (HS980)	B2M knocking out	Knocking out CIITA	—	LN‐521	RPE	[20] (2020)
Knocking out genes except B2M						
hESCs (H9)	—	HLA class I knock down using siRNA and intrabody technology	—	MEF	None	[28] (2011)
hESCs (WIBR3)	—	Knocking out HLA‐A	—	MEF, TCPS	Fibroblasts	[29] (2013)
hESCs (H1)	—	Making HLA‐A, ‐B, ‐C, ‐DR and ‐DQ to be homozygous	—	Not described	EB	[21] (2013)
hiPSCs (strategy 1)	—	Making HLA‐A, ‐B and ‐C to be homozygous& knocking out CIIA	—	iMatrix‐511	CD43^+^ blood cells, cardiomyocytes	[11] (2019)
hiPSCs	—	Knocking out HLA‐B	—	Vitronectin, gelatin, Matrigel, fibronectin	Chondrocyte, endothelial cell, MSC	[30] (2019)
hESCs (HUES8)	—	Knocking out HL‐A, ‐B and ‐C	Knocking in HLA‐G, PD‐L1 & CD47	Geltrex	Endothelial cells, vascular smooth muscle cells	[31] (2019)
hiPSCs	—	Suppression of Bloom syndrome (BLM) gene	—		None	[32] (2019)
Knocking in genes						
hESCs (HUES‐3, HUES‐8)	—	—	Knocking in CTLA4‐Ig, PD‐L1	MEF, Matrigel	Fibroblasts, cardiomyocytes	[33] (2014)
hESCs (H1)	—	—	Knocking in HLA‐G1	MEF	Neural progenitor cells	[34] (2017)
Natural cells						
hiPSCs	—	—	—	MEF	Dendritic cell, HSCs, Antigen‐presenting cells	[35] (2017)

### 2.1 Generation of universal hPSCs by knocking out B2M

**Figure 2:**
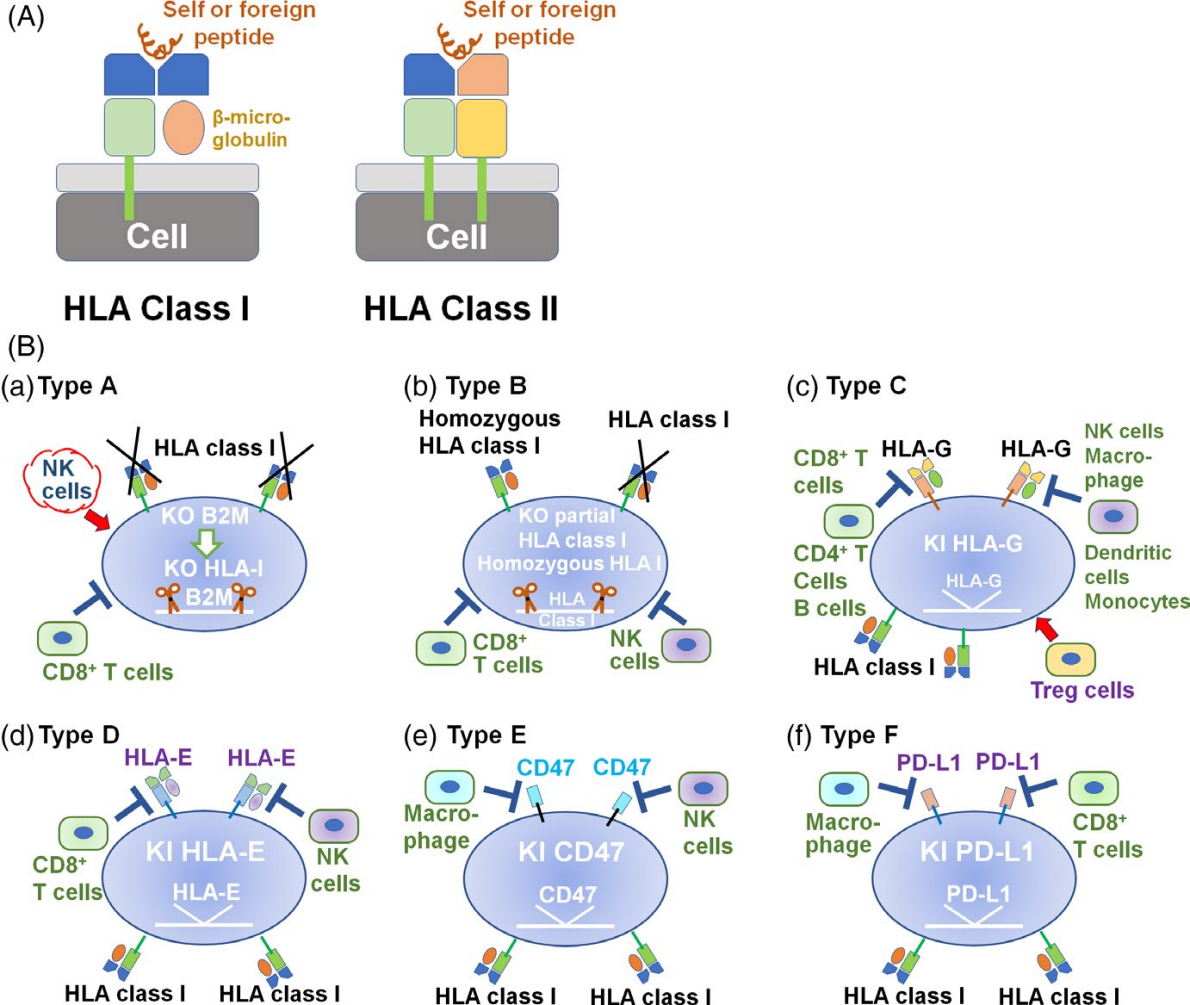
Strategic gene editing of hPSCs to suppress the immune response. A, Schematic structure of HLA class I and class II molecules. β2‐Microglobulin is an essential component of HLA class I molecules. B, Strategic gene editing of hPSCs to suppress the immune response. (a) hPSCs in which B2M is knocked out can escape CD8^+^T‐cell activation but promote NK cell–mediated hPSC killing by inhibiting the missing‐self response. (b) Homozygous hPSCs are generated by knocking out certain HLA class I (HLA‐A, HLA‐B and/or HLA‐C) alleles. (c) hPSCs in which the HLA‐G gene is knocked in inhibit CD8^+^T‐cell activation, macrophage activity and NK cell activity. (d) hPSCs in which the HLA‐E gene is knocked in inhibit CD8^+^T‐cell activation and NK cell activity. (e) hPSCs in which the CD47 gene is knocked in inhibit NK cell activity and macrophage activity. (f) hPSCs in which the PD‐L1 gene is knocked in inhibit CD8^+^T‐cell activation and macrophage activity

Several investigators have designed hPSCs in which B2M is knocked out (Table 2).^5^, ^27^ This is because the B2M protein forms a heterodimer with HLA class I proteins and is required for HLA class I expression on the cell surface (Figure 2A).^11^ Knocking out the B2M gene can restrict an immune response from cytotoxic CD8^+^ T cells by depleting all HLA class I molecules (HLA‐A, ‐B, ‐C, ‐E, ‐F and ‐G), although B2M‐knockout hPSCs become sensitive to natural killer (NK) cell‐mediated killing because they lack the missing‐self response (Figure 2B(a)).^19^

Norbnop et al^25^ generated hematopoietic stem cells (HSCs), megakaryocytes (platelet‐producing cells) and platelets differentiated from B2M‐knockout hiPSCs using clustered regularly interspaced short palindromic repeats (CRISPR)/CRISPR‐associated protein 9 (Cas9) gene editing. HLA‐universal hiPSC‐derived platelets were found to be activated by enhanced CD62P (activated platelet) expression and enhanced aggregation by stimulation with thrombin and arachidonic acid (classic platelet agonists).

One million megakaryocytes derived from B2M‐knockout hiPSCs were intravenously transfused into NSG mice. Two hours and one day after megakaryocyte transfusion, peripheral blood mononuclear cells were collected from the mice, and the expression of human CD41 and CD42b (megakaryocyte and platelet markers) was evaluated by flow cytometry. The results indicated that their engineered platelets could survive in vivo 24 hours after the injection of B2M‐knockout megakaryocytes into mice.

However, there is a concern that cells without HLA class I molecules are targeted by NK cell–mediated cell lysis because they lack the missing‐self response. Furthermore, only 6‐30 platelets are generated from a hPSC‐derived megakaryocyte, whereas 2000‐10 000 platelets are generated from a natural HSC‐derived megakaryocyte. It is necessary to develop a more sophisticated differentiation medium and method for megakaryocyte induction from hPSCs.

Several other researchers have also designed and developed HLA‐universal platelets by disrupting the B2M gene using transcription activator‐like effector nuclease (TALEN) engineering in hiPSCs^22^, ^25^ or RNA interference (RNAi) engineering in hiPSCs and CD34^+^ HSC progenitor cells.^24^, ^25^, ^36^, ^37^

Petrus‐Reurer et al^20^ generated B2M‐knockout hESCs, class II major histocompatibility complex transactivator (CIITA)‐knockout hESCs, and both B2M‐ and CIITA‐knockout hESCs using CRISPR/Cas9 engineering as well as retinal pigment epithelium (RPE) cells differentiated from these gene‐edited cells. All of their gene‐edited cells suppressed both CD4^+^ and CD8^+^ T‐cell activation, but these edited cells enhanced NK cell activation, although they did not show a stronger cytotoxic activity than wild‐type (WT) hESCs. Furthermore, the edited cells were transplanted into a rabbit xenograft model without an immunosuppressive regimen, and the cells suppressed the early rejection response and delayed the production of anti‐human antibodies in the late rejection response.^20^

No presentation of HLA proteins on cells can activate a response by NK cells, and furthermore, there is a risk in the proliferation of cells that are infected by pathogens or a high possibility of tumour development. Therefore, several researchers designed hPSCs in which B2M was knocked out or suppressed, but the edited cells escaped lysis by NK cells.^5^, ^11^, ^23^, ^26^, ^27^

Using an HLA‐1 light‐chain B2M short hairpin RNA (shRNA), Karabekian et al^23^ reported that B2M expression in hESCs was suppressed by lentiviral transduction, where the mRNA levels of B2M were decreased by 90% (and not 100%). These edited hESCs and their differentiated cells (cardiomyocytes) did not activate T‐cell proliferation after treatment with allogenic peripheral blood mononuclear cells. This investigation suggested that the continuous suppression of B2M expression by shRNA might be effective in suppressing not only immune reactions by T‐cell activation but also NK cell responses. This might due to the fact that shRNA expression in the cells did not lead to a complete elimination of B2M expression in hESCs with the advantage of escaping NK cell‐mediating cell killing. Residual HLA expression in HLA‐silenced cells can protect against NK cell–mediated lysis.^23^, ^38^, ^39^ A minimum of 10% residual B2M from the initial B2M expression level in cells prevented the activation of NK cells in these studies.^23^, ^38^, ^39^

However, gene editing using a lentivirus is associated with a risk of inserting genes randomly. One solution to insert the gene safely (the gene of HLA‐1 light‐chain B2M shRNA) is to insert the gene into the safe harbour locus of the *AAVS1* region using CRISPR/Cas9 gene editing.

Xu et al^11^ designed hiPSCs in which B2M was knocked out and HLA‐C was subsequently knocked in by using a customized guide RNA (gRNA) for the CRISPR/Cas9 editing system (HLA‐C‐retained hPSCs) because of the pivotal role of HLA‐C in suppressing NK cells. Furthermore, HLA‐C‐retained hPSCs were also combined with HLA class II‐depleted cells by the targeted knockout of CIITA when necessary.

Mattapally et al^26^ designed hiPSCs (BC‐hiPSCs) in which two key components (B2M and CIITA) were knocked out, which caused the absence of HLA class I and II expression, respectively, using CRISPR/Cas9 genome editing technology. Cardiomyocytes differentiated from BC‐hiPSCs generated extremely low levels of T‐cell activation. Furthermore, BC‐hiPSCs, which express HLA‐G, escaped NK cell recognition and killing in this study.^26^ This is because HLA‐G suppresses NK cell recognition and killing (Figure 2B(c)).

The maternal immune system is tolerant to allogeneic paternal antigens during pregnancy. In this case, the interface between foetal‐maternal blood and foetal tissues, which are composed of cytotrophoblast cells, expresses a low level of HLA class I and II molecules and a high level of CD47, which is a ubiquitous membrane protein and interacts with certain cell surface receptors to escape phagocytosis (Figure 2B(e)).^5^, ^40^ Therefore, Deuse et al^5^ designed hiPSCs (BCC‐hiPSCs) in which B2M and CIITA were both knocked out using CRISPR/Cas9 gene‐editing technology, and subsequently, the CD47 gene was knocked in into hiPSCs using a lentiviral vector (Figure 2B(e)). BCC‐hiPSCs retained their hPSC characteristics and differential abilities. Cardiomyocytes and endothelial cells derived from BCC‐hiPSCs did not express HLA class I and class II molecules based on the flow cytometry assay and escaped the immune response in allogeneic recipients and survived long term without the usage of immunosuppression medicine in humanized mice. In particular, the overexpression of CD47 on BCC‐hiPSCs inhibited NK cell activity and killing potential in vitro and in vivo, and contributed to extremely lower levels of released inflammatory cytokines (IFN‐γ) than that of hiPSCs in which only B2M and CIITA were knocked out.

B2M knockout eliminated the surface expression of all HLA class I proteins. However, these cells were targeted by NK cell lysis, as they lacked the missing‐self response. Gornalusse et al^27^ also reported that a deficiency in the missing‐self response could be prevented not only by CD47 overexpression (Figure 2B(e)) but also by the forced expression of HLA‐E (Figure 2B(d)). HLA‐E was knocked in into hESCs using adeno‐associated virus (AAV)‐mediated gene editing at the B2M locus, where the HLA‐edited hESCs showed no surface expression of HLA‐A, ‐B or ‐C.^27^ The HLA‐edited hESCs and their differentiated cells (RPE cells and HSCs) did not show an allogeneic response by CD8^+^ T cells and resisted lysis by NK cells.^27^ HLA‐E binds to the receptor of NKG2A and CD94, which are expressed in human NK cells.^41^ Therefore, the overexpression of HLA‐E inhibits cell lysis by NK cells. This study demonstrated that HLA‐E expression in hESCs and their differentiated cells that do not express polymorphic HLA class I molecules except for that HLA‐E can prevent the inhibition of the missing‐self response by NK cells. This approach may provide a potential universal or hypoimmunogenic cell source for future clinical applications.

### 2.2 Generation of universal hPSCs by knocking out or knocking down HLA‐A, ‐B and/or ‐C

Torikai et al^29^ developed hESCs in which HLA‐A was knocked out using designer zinc finger nucleases. Although they showed no HLA‐A expression in their genetically edited hESCs, they did not evaluate the allogeneic response by CD8^+^ T cells or lysis resistance by NK cells. Therefore, it is not clear whether it is enough that only one type of HLA, HLA‐A, is knocked out in hPSCs or whether we should knock out multiple types of major types of HLA class I (HLA‐A, HLA‐B and/or HLA‐C) genes to avoid an allogeneic response by CD8^+^ T cells, although the knockout of all major types of HLA class I genes causes lysis by NK cells due to the lack of missing‐self response. In this case, the overexpression of HLA‐G, HLA‐E, CD47 and/or PD‐L1 (Figure 2B(c)‐(f)) can allow escape from NK cell–mediated lysis and T‐cell activation.^26^

On the other hand, Jang et al^30^ knocked out heterozygous HLA‐B from hiPSCs with homozygous HLA‐A utilizing CRISPR/Cas9 genome editing engineering to obtain homozygous HLA‐A hiPSCs with no HLA‐B expression. The HLA‐B‐knockout hiPSCs did not express HLA‐B but expressed pluripotent stem cell markers that were similar to those of the mother hiPSCs. Furthermore, HLA‐B‐knockout hiPSCs exhibited a weaker immune response than the mother hiPSCs according to HLA‐targeted complement‐dependent cytotoxicity assays.^30^ These results indicate that HLA‐edited hiPSCs represent a source of immunocompatible and off‐the‐shelf hiPSCs as well as their differentiated cells for patient therapies.

However, we still need to prepare many hPSC lines with only partial knockout of HLA‐A, HLA‐B or HLA‐C, although edited hPSCs with partial HLA‐A, HLA‐B or HLA‐C expression can escape lysis by NK cells.

Deuse et al^28^ prepared hESCs in which HLA class I molecules were knocked down using intrabody technology to generate hypoimmunogenic hESCs. They demonstrated that their engineered hESCs induced an extensively reduced immune response from T cells, NK cells and macrophages thus extended the survival of the engineered hESCs. However, the knockdown of HLA class I molecules using siRNA and intrabody technology is currently not popular for the preparation of hypoimmunogenic hESCs. This is because it is unclear whether the differentiated cells from hPSCs in which HLA class I molecules are knocked down as well as engineered hPSCs can maintain the absence of HLA class I molecule expression on a time scale of 1‐50 years.

### 2.3 Generation of HLA‐homozygous cell lines by gene editing

HLA‐homozygous cell lines can reduce the number of hPSC cell banks. Therefore, several researchers aimed to generate HLA‐homozygous hPSCs from HLA‐heterozygous hPSCs through a gene editing process (Figure 2B(b)).^11^

**Figure 3:**
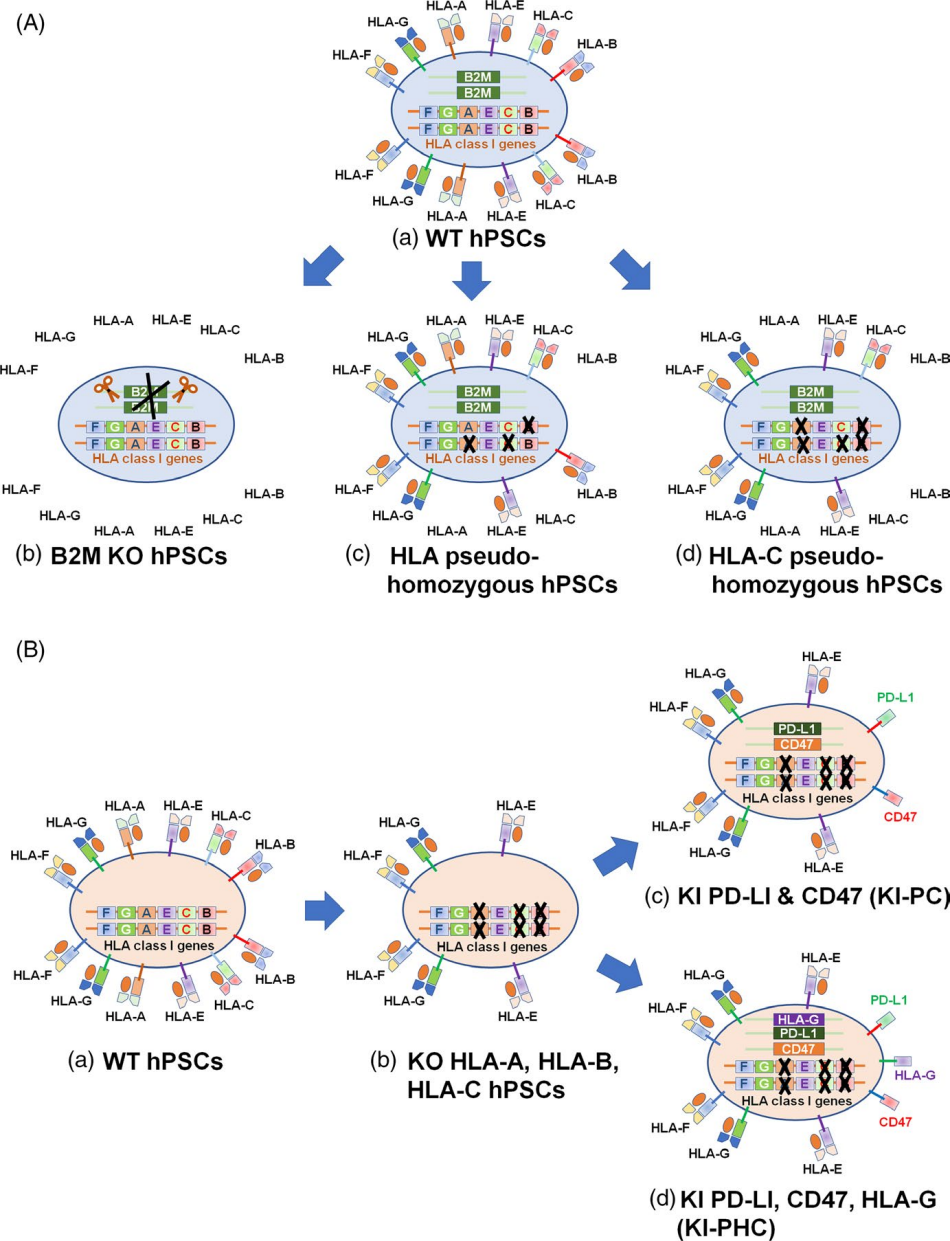
Schematic illustration of HLA pseudo‐homozygous hPSCs and hypoimmunogenic hPSCs generation. A, HLA pseudo‐homozygous hPSC generation. (a) WT hPSCs. (b) B2M‐knocked out hPSCs. (c) HLA pseudo‐homozygous hPSCs by knocking out single alles of HLA‐A, HLA‐B and HLA‐C. (d) HLA pseudo‐homozygous hPSCs by knocking out HLA‐A and hLA‐B as well as single alles of HLA‐C where single HLA‐C alle was remained. B, Hypoimmunogenic hPSCs. (a) WT hPSCs. (b) hPSCs knocked out HLA‐A, HLA‐B and HLA‐C. (c) hPSCs knocked out HLA‐A, HLA‐B and HLA‐C where PD‐L1 and CD47 genes were knocked in. (d) hPSCs knocked out HLA‐A, HLA‐B and HLA‐C where PD‐L1, CD47 and HLA‐G genes were knocked in

All HLA genes share sequence similarity with one another, which increases the risk of targeting non‐specific HLA alleles.^11^ Therefore, Xu et al^11^ prepared a customized gRNA database for the CRISPR/Cas9 system. A total of 2388 gRNAs were found to target single HLA alleles. They also generated hiPSCs with HLA class I haploids by the allele‐specific HLA knockout of normal (heterozygous) hiPSCs as HLA‐homozygous hiPSCs (Figure 3A). Typically, the HLA matching of HLA‐A and HLA‐B but not HLA‐C is evaluated in organ transplantation. Therefore, hiPSCs in which single alleles of HLA‐A and HLA‐B were simultaneously knocked out were generated by CRISPR/Cas9 gene editing. These HLA‐homozygous hiPSCs were differentiated into CD43^+^ (a T‐cell associated marker in this case) blood cells. The differentiated blood cells did not stimulate CD8^+^ T‐cell proliferation. Xu et al further generated pseudo‐homozygous hiPSCs of not only HLA‐A and ‐B but also HLA‐A, ‐B and ‐C (Figure 3A(c)). Their HLA‐A, ‐B, and ‐C pseudo‐homozygous cells escaped T‐cell cytolytic activities. However, a minimum of 50 different types of HLA‐homozygous hiPSCs should be prepared, although only 70% or less of the population is covered^42^ if we consider preparing off‐the‐shelf HLA‐homozygous hiPSCs for clinical usage. Therefore, the generation of pseudo‐homozygous hiPSCs by gene editing is not realistic for clinical applications.

Xu et al^11^ also prepared hiPSCs (hiPSCs‐C) in which HLA‐A and HLA‐B were knocked out, but only a single allele of HLA‐C was knocked out by CRISPR/Cas9 gene editing (Figure 3A(d)). CD43^+^ (a T‐cell associated marker) blood cells differentiated from these hiPSCs were able to escape CD8^+^ T cell–mediated cytolytic activity, which was evaluated from the treatment of CD8^+^ T cells isolated from allogeneic peripheral blood and treated with IFN‐γ obtained from donors who had the same HLA‐C allele.

The hiPSCs‐C cells with HLA‐C expression but no HLA‐A and ‐B expression suppressed NK cell activity.^11^ Interestingly, hiPSCs with HLA‐E but no HLA‐A, ‐B and ‐C (Figure 2B(d)) could not suppress the activation of NK cells in this study. Therefore, one of the major class I genes (class Ia; HLA‐A, ‐B and ‐C) should be retained on HLA‐homozygous hiPSCs to evade NK activation. They also evaluated the graft survival of hiPSCs‐C in vivo using humanized mice.^11^ The number of B2M‐knockout hiPSCs (Figure 3A(b)) quickly decreased after NK cells transplantation, whereas hiPSCs‐C cells (Figure 3A(d)) survived extensively in vivo.

CD8^+^ killer T cells recognize HLA class I molecules for cytotoxic activities, and CD4^+^ helper T cells recognize HLA class II molecules to regulate other immune cells through cytokine release. To obtain immune biocompatibility of hiPSCs‐C, HLA class II‐deficient hiPSCs‐C were also prepared by knocking out the CIITA gene^11^ which leads to cells deficiency in HLA class II molecules because of the lack of antigen‐presentation function via HLA class II molecules. HLA class II mismatched WT hiPSCs and hiPSCs‐C activated the proliferation of CD4^+^ helper T cells, whereas CIITA‐deficient hiPSCs‐C did not stimulate the proliferation of CD4^+^ helper T cells when CD4^+^ helper T cells in which the HLA‐C allele was matched to that of hiPSCs‐C were cocultured with several hPSCs.^11^ These observations indicate that hPSCs, in which the HLA‐A and HLA‐B genes are knocked out and a single allele of HLA‐C as well as CIITA are knocked out using CRISPR/Cas9 gene editing, show no response to CD8^+^ killer T cells, NK cells or CD4^+^ helper T cells.

The number of variant HLA‐C alleles is much smaller than the number of HLA‐A and HLA‐B variant alleles. It has been reported that only 8, 9, 10 and 11 lines of HLA‐C homozygous hPSCs are required for Asian, African American, European American and Hispanic populations, respectively, which cover more than 95% of each population.^11^ Furthermore, only 12 common HLA‐C homozygous hPSCs (HLA‐C *16:01, *12:02, *08:01, *07:02, *07:01, *06:02, *05:01, *04:01, *03:04, *03:03, *02:02 and *01:02) without HLA‐A, ‐B and class II molecules can cover 90% of European American, African American, Asian and Hispanic people.^11^ This is because the matching of more than four alleles from HLA‐A, HLA‐B and HLA‐DR is typically considered for organ transplantation, such as bone marrow transplantation. Therefore, not only universal hPSCs but also HLA‐C‐homozygous hPSCs without HLA‐A, ‐B and class II molecules should be useful in the clinic.

### 2.4 Universal hPSC generation by knocking in some specific genes

Han et al^31^ generated hypoimmunogenic hESCs using CRISPR/Cas9 gene editing in which HLA‐A, ‐B and ‐C as well as CIITA (a HLA class II molecule) were knocked out (Figure 3B). These cells were named KO cells (Figure 3B(b)). Subsequently, PD‐L1 (Figure 2B(f)), HLA‐G (Figure 2B(c)) and CD47 (Figure 2B(e)) were knocked in into the safe harbour locus of *AAVS1* in these HLA‐deficient KO cells (Figure 3B(d)),^43^ which were named KI‐PHC cells. They also prepared KI‐PC cells in which only the PD‐L1 and HLA‐G genes were knocked in as a reference (Figure 3B(c)).^31^ These KI‐PHC cells were based on the fact that T‐cell checkpoint inhibitors of PD‐L1 can protect hPSCs from rejection in humanized mice^33^ and that cells expressing PD‐L1 protect transplanted cells from the immune response by inhibiting PD‐1^+^ macrophages^44^ and PD‐1^+^ NK cells.^45^, ^46^ Furthermore, HLA‐G is an NK cell inhibitory ligand that is typically found at the foetal‐maternal interface during pregnancy via multiple inhibitory receptors.^31^, ^47^, ^48^ The expression of CD47, which is a “do not eat me” signal, can control macrophage activities during the rejection of transplanted cells. These engineered hESCs, KI‐PHC cells and KI‐PC cells, can maintain pluripotency, differentiate into cells derived from the three germ layers, and maintain a normal karyotype as WT hESCs.^31^ The differentiation ability of KI‐PHC and KI‐PC cells into endothelial cells and vascular smooth muscle cells (VSMCs) was found to be similar to that of their WT hESCs. Endothelial cells differentiated from KO cells and KI‐PHC cells with IFN‐γ stimulation, T‐cell proliferation, activation and killing abilities were generated when allogeneic T cells from healthy donors were cocultured with endothelial cells differentiated from WT hESCs, KO cells and KI‐PHC cells.^31^ The total proliferation of T cells as well as CD8^+^ cytotoxic T cells was reduced when cocultured with endothelial cells differentiated from KO hESCs and KI‐PHC cells compared to endothelial cells differentiated from WT hESCs.^31^ CD8^+^ T cells proliferated far less in the presence of endothelial cells derived from KI‐PHC cells compared with endothelial cells derived from KO cells. These results indicate that CD8^+^ T‐cell activation is further reduced by the overexpression of PD‐L1 in KO cells (KI‐PHC cells).

The T‐cell activation markers CD154 (CD40L) and CD69 were significantly reduced when endothelial cells differentiated from KO cells and KI‐PHC cells were cocultured with T cells compared to that endothelial cells derived from WT hESCs were cocultured with T cells. However, there was no T‐cell activation difference between cells cocultured with endothelial cells differentiated from KO cells and those differentiated from KI‐PHC cells.^31^ CD8^+^ T‐cell cytotoxicity was the lowest in the coculture of VSMCs derived from KI‐PHC with CD8^+^ T cells. These results indicate that CD8^+^ T‐cell cytotoxicity is reduced by the expression of PD‐L1 in KO cells, which generates KI‐PHC cells.

Allogeneic NK cells from healthy donors were cocultured with VSMCs derived from KI‐PHC, KO or WT cells, where CD56^+^ NK cells were measured for CD107a (an NK cell activation marker) expression using flow cytometry.^31^ The number of activated NK cells in the coculture with VSMCs derived from KI‐PHC cells was extensively less than that of KO cells and WT hESCs, suggesting that NK cell activity (CD107a expression) as well as NK cell cytotoxicity can be suppressed by the expression of HLA‐G in VSMCs derived from KI‐PHC cells. Furthermore, macrophage engulfment was minimal upon coculture with VSMCs derived from KI‐PHC cells (as measured from red signal detection) compared to when allogeneic macrophages were cocultured with WT cells, KO cells and VSMCs derived from KI‐PHC cells that were labelled with pHrodo‐Red after stimulation with staurosporine to induce apoptosis.^31^ The overexpression of PD‐LI as well as CD47 seems to be the main reason for which macrophage engulfment on VSMCs derived from KI‐PHC cells was reduced. The hypoimmunogenic hESCs, KI‐PHC cells, displayed minimum immune activation and killing by allogeneic NK cells or T cells and further showed minimal engulfment by allogeneic macrophages.^31^

Rong et al^33^ generated gene‐edited hESCs (PC‐hESCs) that constructively express PD‐L1 and cytotoxic T lymphocyte antigen 4 (CTLA4)‐immunoglobulin (Ig) even after differentiation into fibroblasts and cardiomyocytes. CTLA4 has high binding affinity to CD86 and CD80, which are the primary signalling pathways involved in the activation of T cells. Then, a fusion protein of CTLA4 and Ig was designed to inhibit the T cell–mediated immune response.^33^, ^49^ PD‐L1 has high binding affinity to programmed cell death 1 (PD‐1), which is displayed on T‐cell surfaces where the interaction between PD‐L1 and PD‐1 leads to the inhibition of T‐cell activities.^33^, ^50^ The differentiated cells from PC‐hESCs did not generate an immune response when transplanted into humanized mice, whereas the genetically non‐edited original hESCs were extensively rejected in humanized mice. The overexpression of PD‐L1 leads to inhibition of the T‐cell activation pathway, and the expression of CTLA4‐Ig disrupts the T‐cell costimulatory signalling pathway. The expression of both PD‐L1 and CTLA4‐Ig on hESCs is necessary for immune protection, and the expression of one of these genes is insufficient for immune protection.^33^ Notably, knocking out only PD‐L1 and CTLA4‐Ig suppressed the T‐cell response but did not suppress the NK cell killing response nor the macrophage phagocytosis response in this study. However, these results suggest that knocking in specific genes related to the inhibition of the immune response in hPSCs can also be used to generate universal or hypoimmunogenic hPSCs without inducing systemic immune suppression.

It is known that maternal immune tolerance to the foetus is partially based on HLA‐G expression in maternal tissue, which can be detected in placental cells during pregnancy. Therefore, Zhao et al^34^ prepared hESCs (G‐hESCs) in which HLA‐G was overexpressed using a lentiviral vector (Figure 2B(c)). Their engineered hESCs maintained hESC characteristics, including pluripotent protein expression and differentiation ability into cells derived from the three germ layers. The hESCs overexpressing HLA‐G and their differentiated cells into neural progenitor cells showed some immune tolerance protection against CD8^+^ T cells and NK cells.^34^ However, immune tolerance ability was not significant based on their results. Furthermore, lentiviral vector infection may induce the generation of malignant cancer cells because of randomly incorporated cDNA into host cells. Therefore, more sophisticated gene editing methods, such as CRISPR/Cas9 gene editing, should be considered in this area of research.

## 3 CONCLUSION AND FUTURE PERSPECTIVES

Hypoimmunogenic hPSCs in which only a single HLA‐C allele is expressed but HLA‐A, HLA‐B and CIITA are knocked out, prepared using CRISPR/Cas9 gene editing by Xu et al,^11^ and hPSCs in which HLA‐A, HLA‐B, HLA‐C and CIITA are knocked out and PD‐L1, HLA‐G and CD47 are knocked in at the *AAVS1* safe harbour locus, prepared using CRISPR/Cas9 gene editing by Han et al,^31^ are currently the most promising universal and hypoimmunogenic hPSCs.

For stem cell therapy in the future, the genome editing process must follow the regulatory guidelines for the preparation of clinical‐grade gene‐edited hPSCs.^51^ It is also necessary to select a hPSC line from a donor with blood type O to minimize the immune response against ABO antigens. The genomic integrity of edited hPSCs must be determined from not only karyotyping but also whole‐exome sequencing (WES) analysis, which identifies several single nucleotide variations (SNVs) as well as indels, including on‐target and off‐target indels, on the targeted HLA genes. Although several humanized murine models have been developed to evaluate the immunogenicity of transplanted cells, these humanized mice are still limited to recapitulating a full immune response in humans. It should be extremely important to develop more plausible, realistic and less expensive humanized mouse models for the evaluation of graft versus host disease (GVHD) and the mechanism of GVHD when the cells are transplanted into patients. Long‐term usage of immune‐suppressive medicine leads to allograft rejection. In particular, general immunosuppressant regimens are sometimes toxic to patients with chronic disabling inflammation diseases. Furthermore, chronic immune suppression using immune‐suppressive medicine greatly enhances the risk of cancer and viral infections. Therefore, universal hPSCs, homozygous hPSCs and their differentiated cells are valuable cell sources as off‐the‐shelf cell lines in future stem cell‐based therapy for clinics.

## CONFLICTS OF INTEREST

The authors declare no conflicts of interest.

## AUTHOR CONTRIBUTIONS

Qingsong Ye collected articles and advised the manuscript. Tzu‐Cheng Sung prepared tables and figures. Jen‐Ming Yang prepared tables and figures. Qing‐Dong Ling and Yan He advised the manuscript. Akon Higuchi designed the research project and idea, summarized data and wrote manuscript. All authors read and approved the final version of the manuscript as submitted.

## Data Availability

Data sharing is not applicable to this article as no new data were created or analysed in this study.
